# Ordinal Time Series Forecasting of the Air Quality Index

**DOI:** 10.3390/e23091167

**Published:** 2021-09-04

**Authors:** Cathy W. S. Chen, L. M. Chiu

**Affiliations:** Department of Statistics, Feng Chia University, Taichung 407, Taiwan; m0703432@mail.fcu.edu.tw

**Keywords:** ARX-GARCH model, autoregressive logistic regression, artificial neural network, machine learning, support vector machine, multi-class classification, one-step-ahead forecast, training and validation

## Abstract

This research models and forecasts daily AQI (air quality index) levels in 16 cities/counties of Taiwan, examines their AQI level forecast performance via a rolling window approach over a one-year validation period, including multi-level forecast classification, and measures the forecast accuracy rates. We employ statistical modeling and machine learning with three weather covariates of daily accumulated precipitation, temperature, and wind direction and also include seasonal dummy variables. The study utilizes four models to forecast air quality levels: (1) an autoregressive model with exogenous variables and GARCH (generalized autoregressive conditional heteroskedasticity) errors; (2) an autoregressive multinomial logistic regression; (3) multi-class classification by support vector machine (SVM); (4) neural network autoregression with exogenous variable (NNARX). These models relate to lag-1 AQI values and the previous day’s weather covariates (precipitation and temperature), while wind direction serves as an hour-lag effect based on the idea of nowcasting. The results demonstrate that autoregressive multinomial logistic regression and the SVM method are the best choices for AQI-level predictions regarding the high average and low variation accuracy rates.

## 1. Introduction

Monitoring air quality is important for human health and the environment. The well-known air quality index (AQI) presents the status of daily air quality and shows the degree of air pollution and how certain factors affect people’s health. This index covers five major air pollutants: (1) ground-level ozone; (2) particle pollution (known as particulate matter, including $PM_{2.5}$ and $PM_{10}$); (3) carbon monoxide; (4) sulfur dioxide; (5) nitrogen dioxide. The higher the AQI level is, the greater is the degree of air pollution, and the more serious its impact is on the health of human beings. The U.S. Environmental Protection Agency (EPA) typically employs an AQI value of 100 as the benchmark for air quality to help protect people’s health. With reference to U.S. standards, Taiwan’s AQI includes an 8-h moving average of ozone, an hourly average of ozone, a 12-h weighted average of $PM_{2.5}$, a 12-h weighted average of $PM_{10}$, an 8-h average of carbon monoxide, hourly average of sulfur dioxide, and hourly average of nitrogen dioxide. With a total of seven indicators, the most serious index value then denotes the value of AQI. The U.S. EPA classifies AQI values into six levels, and each level corresponds to different health problems; see Table 1.

Predicting AQI classes plays an important role in issuing health alerts when AQI levels exceed the specified levels. This study thus aims to build a forecasting system based on the level of AQI rather than AQI values. Categorical time series can be either nominal or ordinal. The time series of AQI levels is ordinal in the sense that the class increases as the AQI interval value increases. Some studies in the literature focus on forecasting AQI values or individual pollutant concentrations based on neural network, mode decomposition, and ARIMA (autoregressive integrated moving average) models; e.g., [1,2,3,4,5]. Alternatively, Liu et al. [6] propose a zero-one-inflated bounded Poisson model for air quality level data, while Kim [7] employs a generalized linear model for the ozone level. Our study mainly predicts multi-level AQI classifications, which help forecast qualitative AQI at time *t* based on information up to t−1. With respect to the prediction, we should not utilize current information, especially as AQI includes the pollutants $SO_{2}$, $NO_{2}$, $O_{3}$, CO, $PM_{10}$, and $PM_{2.5}$. We instead consider three weather covariates and seasonal variables that could influence the change in AQI, including daily accumulated precipitation (PRE), daily average temperature (TEM), and daily wind direction (WD). Henry et al. [8] use nonparametric regression to determine the relationship between the concentrations of air pollutants and WD. We utilize an hour-lag effect of WD, which is widely used by meteorologists for nowcasting—that is, information of the covariate WD is available up to time (t+m−1), where *m* is the hour difference between AQI and WD.

As AQI relates to its own historical information [9], we utilize the following statistical modeling and machine learning approaches with the three weather covariates and seasonal dummy variables for the predictions. First, we employ an autoregressive model with the above-mentioned weather covariates (commonly called exogenous variables) and GARCH (generalized autoregressive conditional heteroskedasticity) errors if heteroscedasticity exists in the time-series data; see So et al. [10] and Chen et al. [11]. The ARCH and GARCH models originally appear in Engle [12] and Bollerslev [13] and have been widely used in modeling the conditional second-moment properties of time-series data. We observe that the time series of AQI values exhibit conditional heteroskedasticity and autocorrelation in the squared series, which are said to have ARCH effects. We then predict one-step-ahead AQI values and convert them into the AQI level based on this proposed model, which we name the ARX-GARCH model. Here, “X” stands for those covariates and seasonal variables.

Second, we employ an autoregressive multinomial logistic regression for the AQI ordinal response variable, for which this model includes the lag-1 AQI value, the weather covariables, and seasonal variables. This model is an excellent tool for analyzing binary and ordinal data, allowing us to estimate effects and to make predictions [14]. We call this model the AR logistic regression for short hereafter. Some popular classifiers to forecast pollution are neural networks [15] and support vector machines (SVM) [16,17]. SVM, a type of machine learning technique, is a supervised learning model with related learning algorithms that analyze data used for binary and multi-level classifications.

Third, we use the SVM method with the same covariates as in the AR logistic regression for classification and prediction. Liu et al. [17] utilize the current effects of $PM_{2.5}$, $PM_{10}$, $SO_{2}$, CO, $NO_{2}$, and $O_{3}$ as input variables. On the contrary, our study excludes these current effects for the forecast purpose. We instead incorporate the lag-1 AQI value in the forecasting models since AQI covers the five major air pollutants, which simultaneously include many lags of the major pollution concentrations.

Fourth, we employ the neural network autoregression with exogenous variable (NNARX) model, which is capable of recognizing, classifying, or predicting datasets. Applications of autoregressive neural network modeling have been successfully predicting time-series datasets; e.g., Hertz et al. [18] and Hyndman and Athanasopoulos [19].

This study encounters several missing values in AQI, such as accumulated precipitation, daily average temperature, and wind direction. In order to preserve the characteristics of the time series, we do not remove any missing values directly for information loss and instead adopt *k*-nearest neighbors as proposed by Cover and Hart [20] to fill in the missing values; see Torgo [21] for the *k*-Nearest Neighbors (knn) algorithm. Hence, we implement the knn imputation to fill in the missing value under different *k* values with different variables to make the mean absolute error (MAE) and root mean squared error (RMSE) as small as possible.

The rest of this study runs as follows. Section 2 introduces the models for forecasting ordinal time series—the AQI levels. Section 3 shows the data description and provides information about training/validation of the methodology in order to assess the forecast performance. Section 4 provides the AQI level forecasts and evaluates forecast accuracy. Finally, Section 5 offers concluding remarks.

## 2. Models and Methods

To predict the one-step-ahead AQI level, we describe the four proposed models for the daily prediction as follows. Let $Y_{t}$ be the daily AQI value, $Y_{t-1}$ is the previous day’s AQI value, and $\left(X_{1,t},X_{2,t},X_{3,t}\right)$ are respectively PRE, TEM, and WD. Let $Y_{t}^{*}$, t=1,…,n be the observed AQI level at time *t*, with the following *r* possible outcomes:

$$\begin{array}{c} Y_{t}^{*}=\{\begin{array}{cc} j & \;\mathrm{if}\;\mathrm{the}\;j\mathrm{th}\;\mathrm{level}\;\mathrm{is}\;\mathrm{observed}\;\mathrm{at}\;\mathrm{time}\;t,\\ 0 & \;\mathrm{otherwise}, \end{array} \end{array}$$

for j=1,…,r. The proposed methods consider a seasonal or periodic pattern to improve the prediction via seasonal dummy variables or a monthly indicator.

Model 1: The ARX model with GARCH errors

(1) $$Y_{t}=\phi_{0}+\phi_{1}Y_{t-1}+f(X_{1,t-1},X_{2,t-1},X_{3,t+m-1})+SN_{t}+a_{t},$$

$$a_{t}=\sigma_{t}\varepsilon_{t},\;\varepsilon_{t}\overset{iid}{∼}St\left(\nu ,r\right)$$

(2) $$\begin{array}{ccc} \sigma_{t}^{2} & = & \alpha_{0}+\alpha_{1}a_{t-1}^{2}+\beta_{1}\sigma_{t-1}^{2}, \end{array}$$

(3) $$f(.)=\gamma_{1}X_{1,t-1}+\gamma_{2}X_{2,t-1}+\gamma_{3}X_{3,t+m-1},$$

$$SN_{t}=\beta_{s1}D_{s1,t}+\beta_{s2}D_{s2,t}+\ldots +\beta_{s11}D_{s11,t},$$

$$\begin{array}{ccc} D_{si,t} & = & \left\{ \begin{array}{cc} 1 & \mathrm{if}t\;\mathrm{is}\;\mathrm{in}\;\mathrm{month}\;i\\ 0 & \mathrm{otherwise}, \end{array}\right. \end{array}$$

where $a_{t}$ is the error term; $\varepsilon_{t}$ follows the skewed Student-t distribution by Hansen [22] since the data are non-normal; and St(ν, *r*) is a skewed Student-t distribution with shape parameter ν and skewness parameter *r*. Here, (1) and (2) are respectively the mean equation and the volatility equation. The lag *m* of $X_{3,t+m-1}$ denotes the time difference in hours between AQI and WD. It is based on the idea of nowcasting. In order to guarantee stationarity and positiveness in volatility, we restrict the parameters as:

$$\begin{array}{ccc} S1 & : & |\phi_{1}|<1\\ S2 & : & \alpha_{0}>0,\;\alpha_{1},\beta_{1}\geq 0,\;\alpha_{1}+\beta_{1}<1. \end{array}$$

As the ARX-GARCH models in (1) are designed for continuous dependent variables, we obtain the one-day-ahead forecast based on the ARX-GARCH model and then convert the forecasts into the AQI level as in Table 1.

Latent variable models with observed ordinal variables are particularly useful for analyzing the AQI levels. Next, we consider an AR logistic regression for $Y_{t}^{*}$.

Model 2: The AR logistic regression 

The conditional probability for the AQI level at day *t* is:

(4) $$\begin{array}{ccc} & & \mathrm{Pr}(Y_{t}^{*}=i|Y_{t-1},X_{1,t-1},X_{2,t-1},X_{3,t+m-1})=\\ & & \frac{\exp(g_{i}(Y_{t-1},X_{1,t-1},X_{2,t-1},X_{3,t+m-1},SN_{t}))}{1+\sum_{j=1}^{r-1}\exp(g_{j}(Y_{t-1},X_{1,t-1},X_{2,t-1},X_{3,t+m-1},SN_{t}))}, \end{array}$$

where $g_{i}\left(.\right)$ is a linear function with i=1,…,r−1. Apart from this model, Weiß [23] and subsequently Liu et al. [6] propose two new methods for dealing with ordinal time series; e.g., the rank-count approach for time series modeling.

Model 3: The SVM classification method

SVM classification is an optimization problem, which is a machine learning algorithm for the dependent variable, isolating the group to which it should belong. If the subset trained in two dimensions is a linearly separable set, then it means that the whole set itself is linearly separable. We also can use SVM for classifying a non-linear dataset and conduct the classification by projecting the dataset into a higher dimension in which it is linearly separable. We consider multi-dimensions, where the dividing line becomes a separating hyperplane and separates into more than two different levels. The hard-margin must be met so that the distance between the separating hyperplane and each group is equal; in other words, the margins are maximized. We use the SVM method, and the hyperplane h(X) is:

(5) $$\begin{array}{ccc} h(X) & = & \alpha +\phi_{1}Y_{t-1}+f(X_{1,t-1},X_{2,t-1},X_{3,t+m-1})+\beta_{4}M_{t}, \end{array}$$

$$\begin{array}{ccc} M_{t} & = & \left\{ \begin{array}{cc} i & \mathrm{if}\;t\;\mathrm{is}\;\mathrm{in}\;\mathrm{month}\;i\\ 0 & \mathrm{otherwise}, \end{array}\right. \end{array}$$

where f(.) is the same as in Equation (3). The algorithm creates a line or a hyperplane that separates the data in the training (or learning) period into classes. Each hyperplane h(X) that is trained by the learning period divides the validation period into specific levels and gives a score based on their proximity to the hyperplane. We execute the final step of classifications by assigning the validation period to the level from which they obtain the highest score.

Model 4: We consider the NNARX model, feed-forward networks, and two hidden layers and use the notation NNARX(1,2). This model includes the lag-1 AQI value and $\left\{X_{1,t-1},X_{2,t-1},X_{3,t+m-1}\right\}$ as exogenous variables. The NNARX model with hidden layers achieves greater forecasting accuracy, but the cost is a large computation time as the number of hidden layers increases. 

## 3. Data Collection

Particulate pollutants originating from the Asian continent, in particular eastern China, are often carried to Taiwan along with the monsoons; see Hsu et al. [24]. This means the WD covariate plays a significant role in the prediction of the AQI level. The other pollutants in Taiwan mainly come from domestic combustion such as the burning of fossil fuels (industrial emissions). The remainder comes from traffic emissions and other air pollution sources. The industrial centers and business parks along the northern and western coasts of Taiwan are surrounded by high mountains, which lead to poor avenues of dispersement and can trap pollutants [24].

Chen et al. [25] support the causal relationship between weekly influenza cases and weekly adjusted accumulative $PM_{2.5}$ in the central and southern regions of Taiwan for various age groups. Traffic-related emissions and coal combustion are the sources of toxic metals in $PM_{10}$ and $PM_{2.5}$, respectively, leading to high risks of cancer [24]. Tseng et al. [26] provide epidemiological evidence that more than 50% of all patients with lung cancer had never smoked and present a positive association between lung cancer and $PM_{2.5}$ exposure in Taiwan, while Tang et al. [27] demonstrate that air pollution in Taiwan, represented by $PM_{2.5}$ or PSI (Pollutant Standards Index), moderately correlates with the development of atopic dermatitis in adults.

**Figure 1:**
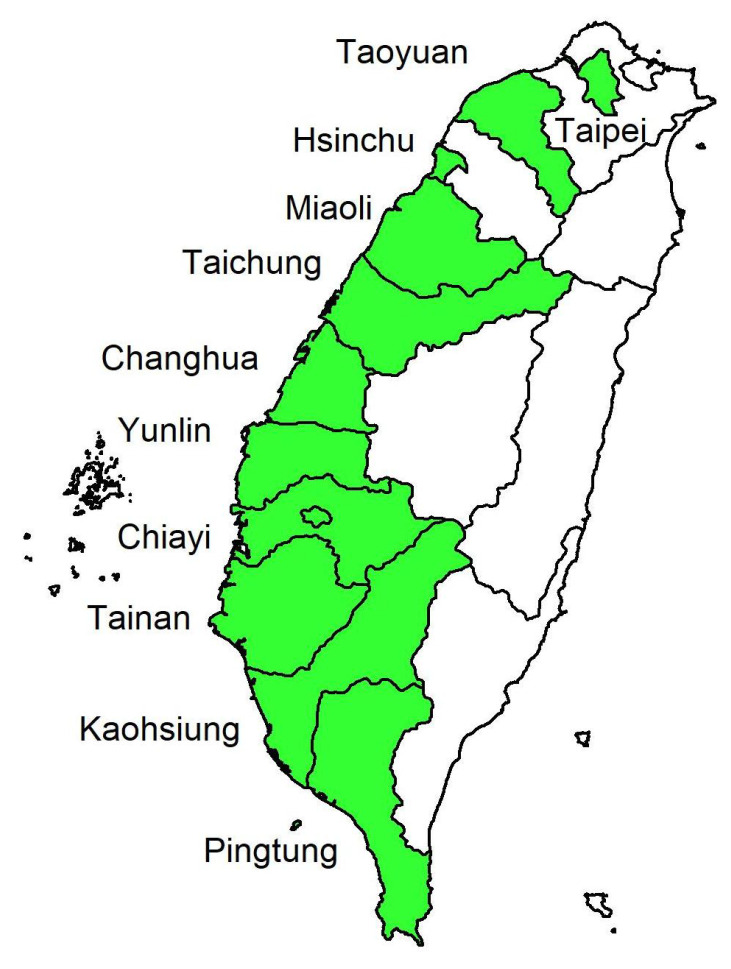
Geographical locations of the 16 sites in Taiwan. Taipei: site 1; Taoyuan: site 2; Hsinchu: site 3; Miaoli: site 4; Taichung: sites 5 and 6; Changhua: site 7; Yunlin: site 8; Chiayi: sites 9 and 10; Tainan: sites 11 and 12; Kaohsiung: sites 13 and 14; Pingtung: sites 15 and 16.

**Figure 2:**
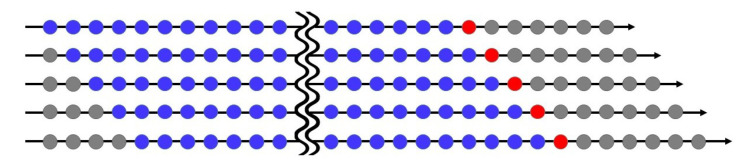
The rolling window approach.

Wide differences in air pollution trends have existed between northern and southern Taiwan for decades [26]. We thus investigate daily AQI from the monitoring stations of 16 cities/counties in Taiwan from north to south on the country’s west coast, whose names are listed in Table 2 and their geographical locations appear in Figure 1. Our dataset covers a total of 1227 observations from 30 November 2016 to 9 April 2020 at each monitoring station and includes daily data for AQI, $PM_{2.5}$, PRE, TEM, and WD for the 16 cities/counties from EPA, Executive Yuan, Taiwan. The training period includes 852 observations (from 30 November 2016 to 31 March 2019), and we use the last 375 days (from 1 April 2019 to 9 April 2020) as the validation period via a rolling window approach. In order words, we choose a rolling sample size of 852 for parameter estimation (or classification) in the training period and forecast the one-day-ahead AQI level for the validation period. The mechanism of the rolling window approach appears in Figure 2. We measure WD in units from $0^{\circ }$ to $360^{\circ }$. When the wind speed is ≤ 0.2 m/s, we record WD as $0^{\circ }$ and the north wind as $360^{\circ }$. As the variable WD (in degrees) itself does not have any physical meaning, we classify wind directions in sixteen sectors in Table 3, making it a quantitative variable.

Some missing values are present in the dataset that are often encountered in practice. The percentages of missing values for AQI are between 0.97% and 2.09%. A small portion of missing values also occurs in the weather covariates. When we deal with a tiny proportion of missing values, we still need to tackle this problem in time series data. We employ the book-associated R package called “DMwR” in Torgo [21] to impute the missing values. Due to limited space, we summarize how we handle missing value imputation below.

(1) PRE or TEM with missing values: If PRE (TEM) is missing at day *t*, then we select the valid data of the nearest station to impute the missing value.
(2) AQI with missing values: We use k=4 in the knn algorithm in terms of the smallest MAE and RMSE to impute the missing AQI value. We first identify the four days with PRE, TEM, WD, $PM_{2.5}$, and seasonal dummy values closest to the day with missing AQI, and the missing AQI is then substituted by the weighted average of the AQI values of these four days. The weights decrease as the distance of the case to its neighbor lengthens. We use a Gaussian kernel function to obtain the weights from the distances and refer for a detailed description of the distance to Torgo [21] on pages 61–62.
(3) WD with missing values: We again use k=4 in the knn algorithm to impute the missing WD value. We first identify the four days with PRE, TEM, AQI, $PM_{2.5}$, and seasonal dummy values closest to the day with missing WD, and the missing WD is then substituted by the weighted average of the WD values of these four days.

In order to better understand the characteristics of our datasets, Table 2 displays descriptive statistics of AQI values for each site, which include mean, standard deviation (std), minimum, maximum, and *p*-values of the ARCH test. All averages of AQI values are above 50 except for Hengchun (44.8 at site 16), as Hengchun Peninsula in Pingtung County is considered to have clean air. Kaohsiung and Pingtung (sites 13, 14, and 15) have the three worst AQI values among the observed sites in this study. The *p*-values of the Kolmogorov–Smirnov test ($H_{0}$: The series is from a normal distribution) are all less than 0.0001, indicating that the AQI data do not follow a normal distribution.

The ARCH test is a Lagrange multiplier test to assess the significance of ARCH effects. A small *p*-value indicates rejection of the null hypothesis ($H_{0}$: There is no ARCH effect) in favor of the alternative. All *p*-values of the ARCH test are less than 0.0001, which supports the existence of ARCH effects. Heteroscedasticity refers to residuals for an AR model that do not have a constant variance. Therefore, we need to specify the GARCH effect and non-normal errors instead of regular AR models. Table 4 lists descriptive statistics for two weather covariates: PRE and TEM. The standard deviation of “PRE” increases toward the south in general.

**Figure 3:**
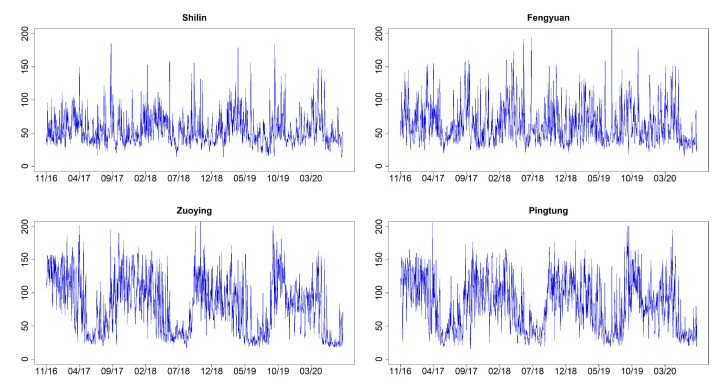
Time series plots of daily AQI values for Shilin, Fengyuan, Zuoying, and Pingtung (sites 1, 6, 13, and 15) from 30 November 2016 to 9 April 2020.

**Figure 4:**
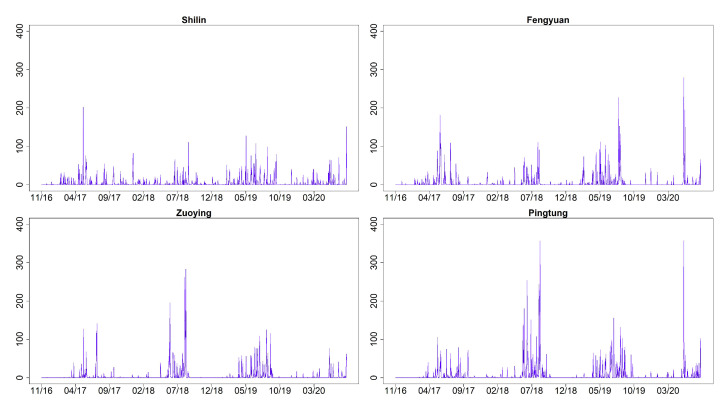
Time series plots of daily PRE for Shilin, Fengyuan, Zuoying, and Pingtung (sites 1, 6, 13, and 15) from 30 November 2016 to 9 April 2020.

**Figure 5:**
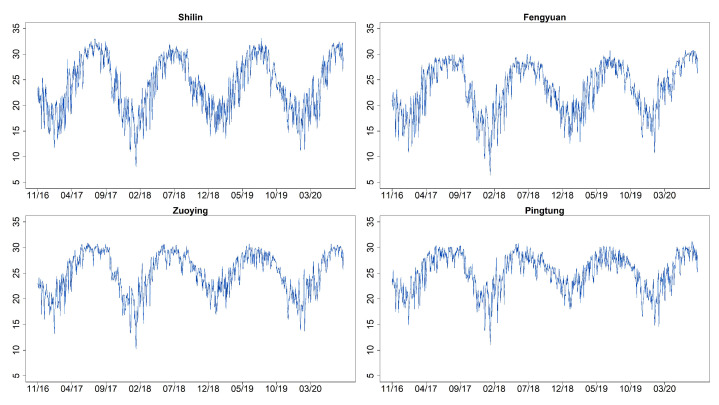
Time series plots of daily TEM for Shilin, Fengyuan, Zuoying, and Pingtung (sites 1, 6, 13, and 15) from 30 November 2016 to 9 April 2020.

**Figure 6:**
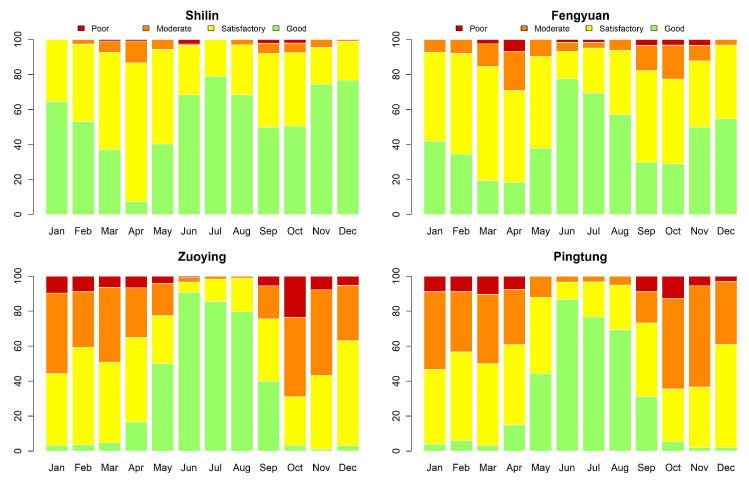
Monthly AQI levels for Shilin, Fengyuan, Zuoying, and Pingtung (sites 1, 6, 13, and 15) from 30 November 2016 to 9 April 2020.

Figure 3 shows the time series plots of daily AQI values (imputation for missing datapoints) for Shilin, Fengyuan, Zuoying, and Pingtung (sites 1, 6, 13, and 15 given in Table 2). These four monitoring stations represent the north, center, and south of Taiwan. Figure 4 and Figure 5 illustrate their related daily PRE and TEM in the same four sites. The time series plots of AQI present obvious seasonal patterns with a gradual decrease in May each year. AQIs are relatively low in June and July and gradually increase again until October. The pattern denotes that AQI is lower in summer than in other seasons, and the worst season is winter. We clearly notice that the rainy season is from May to September, and the highest amount of daily accumulated precipitation appears in July and August. Other cities/counties also present similar seasonal patterns. We observe that heavy rain is more likely to occur in the central and southern regions. In addition, the average temperature also shows a seasonal pattern. There are no sixth-level AQI values (301–500) in this study. We present the relative frequencies of AQI levels for each month based on the four monitoring stations noted above in Figure 6. We discover that a larger proportion of the first level (good) appears during summer. “Moderate” and “poor” air quality occurs in summer in a small proportion and then at a higher proportion in spring and winter. We scrutinize larger proportions in levels 2 and 3 (AQI >100) in Zuoying and Pingtung, which represent south Taiwan.

## 4. Results

We predict the one-day-ahead AQI level by using a rolling window approach with a hold-out set: 375 days, which cover just a bit more than one year. We evaluate forecast performance for air quality levels, which include two levels and four levels based on four models: (1) the ARX-GARCH model; (2) the AR logistic regression; (3) the SVM method; (4) the NNARX model.

We add 11 monthly dummy variables as covariates in the ARX-GARCH model and AR logistic regression since the AQI time series presents a seasonal pattern. We use 1 to 12 values of the month indicator variable in the SVM method and NNARX model, because this setup improves the accuracy rate in the AQI-level prediction. We estimate the model parameters and the one-day-ahead forecasts by using the R package “rugarch” [28] for Model 1 (ARX-GARCH model). For the AR logistic regression and SVM method, we use the R package “MASS” [29] and the “e1071” package [30], respectively. For the NNARX model, we employ the R package “neuralnet”.

The categorization used for the two-level forecast is based on the threshold 100 (1st level: AQI ≤100; 2nd level: AQI >100). Table 5 presents the correct classification rates for two-level predictions. The accuracy rates of all sites are greater than 81% for the SVM method. Most accuracy rates by the AR logistic and NNARX models are over 80%, except for site 15 at 78.13%, while the NNARX model has accuracy rates of 79.73% and 78.40% for sites 13 and 15, respectively. The ARX-GARCH model does not predict sites 8, 13, 14, and 15 very well.

**Figure 7:**
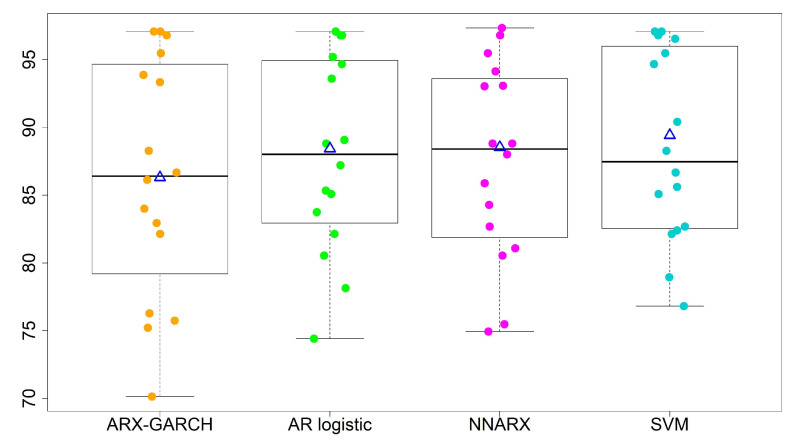
Forecasting performance for two-level classifications. “△”denotes the average of accuracy rates from all 16 sites.

**Figure 8:**
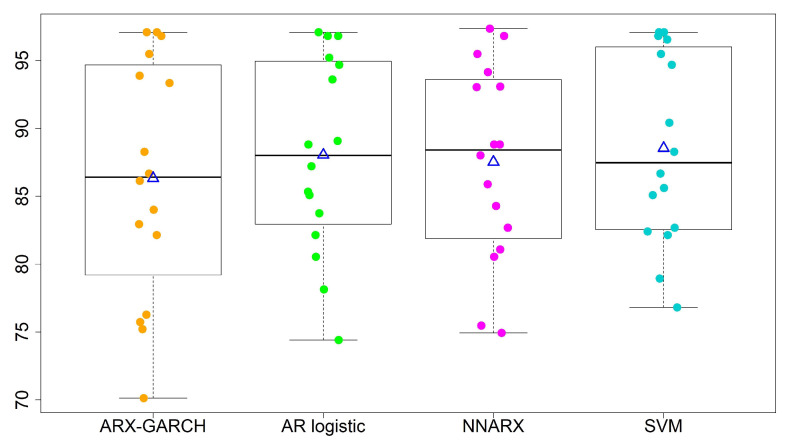
Forecasting performance for four-level classifications. “△” denotes the average of accuracy rates from all 16 sites.

We refine the second category into three levels: (i) 101–150 slightly polluted; (ii) 151–200 moderately polluted; (iii) 201–300 heavily polluted. When we turn to multi-level forecasting, Table 6 illustrates the percentage accuracy, average, standard deviation (std), and coefficient of variation (CV) of the four models. We show boxplots (a five-number summary with minimum, 1st quantile, median, 3rd quantile, and maximum) for the two-level and four-level predictions in Figure 7 and Figure 8. The notation △ stands for the average of accuracy rates from all 16 sites for each model. We summarize the results as follows.

1. The overall performances of the AR logistic regression, SVM, and NNARX are similar. Most accuracy rates by the three models are over 80%, except for sites 13 and 15. The SVM classification method has the highest average accuracy rate (88.53%) and the lowest standard deviation (7.00%) in the four-level predictions. The SVM method and AR logistic regression provide the lowest CV values among the four forecasting techniques. A lower CV is favored because it provides the most optimal forecasting performance with low variability, but a high average accuracy rate.
2. The prediction performance is relatively low at sites 13 and 15, and so we need to scrutinize the time series data carefully. We perceive that the major misspecified levels occur in October. The three classification models (AR logistic regression, SVM, and NNARX) tend to misspecify the rare level.
3. The lag-1 weather covariates of PRE and TEM with the hour-lag effect of WD are able to generate a reliable prediction level. We also consider wind speed and weekly/monthly weighted moving averages of AQI as extra covariates. However, these covariates do not improve forecast accuracy. Therefore, we do not include their results in the paper.
4. The ARX-GARCH model is for quantitative forecasting purposes. The classification of wind directions in the sixteen sectors may not be suitable as an explanatory variable for this model.

## 5. Conclusions

This study predicts one-day-ahead AQI levels for 16 cities/counties in Taiwan based on training/validation of the methodology set up herein and examines forecast accuracy by considering the ARX-GARCH, AR logistic regression, SVM, and NNARX models. We assess the accuracy of these forecasting models by a rolling window approach. These models relate to lag-1 AQI values and previous day weather covariates (PRE and TEM), while WD is a time-lag effect based on the idea of nowcasting. The results demonstrate that AR logistic regression and the SVM method are the best choices for AQI-level predictions regarding the high average and low variation accuracy rates among the four forecasting techniques. This information can greatly help the authorities to take proper action for AQI-level prediction.

There are many other established tools for ordinal time series classification, and it is worthwhile to predict ordinal time series using two other recently developed methods: the distance-based approach and the bounded-count method. We leave these new techniques for forecasting ordinal time series as a future research direction.

## Figures and Tables

**Table 1:** AQI’s six levels based on the U.S. EPA, where each level corresponds to different health problems.

Value	Color	AQI Levels of Health Concern
0–50	Green	Good
51–100	Yellow	Satisfactory
101–150	Orange	Moderately
151–200	Red	Poor
201–300	Purple	Very poor
301–500	Maroon	Hazardous

**Table 2:** Descriptive statistics of daily AQI values.

Site	City/County	Mean	Std	Min	Max	ARCH Test (*p*-Value)
1	Shilin, Taipei	54.888	23.799	13	185	<0.0001
2	Taoyuan, Taoyuan	54.925	24.443	15	197	<0.0001
3	Dongqu, Hsinchu	57.873	27.444	12	203	<0.0001
4	Miaoli, Miaoli	61.197	25.893	13	172	<0.0001
5	Xitun, Taichung	67.271	32.275	19	190	<0.0001
6	Fengyuan, Taichung	62.861	29.702	15	207	<0.0001
7	Changhua, Changhua	65.298	31.092	15	190	<0.0001
8	Douliu, Yunlin	80.409	38.347	1	195	<0.0001
9	Xingang, Chiayi	74.014	34.615	6	179	<0.0001
10	West, Chiayi	75.400	36.941	16	179	<0.0001
11	West Central, Tainan	74.512	37.539	17	195	<0.0001
12	Xinying, Tainan	74.445	34.831	15	179	<0.0001
13	Zuoying, Kaohsiung	81.031	42.767	17	210	<0.0001
14	Quian, Kaohsiung	79.332	41.196	17	197	<0.0001
15	Pingtung, Pingtung	81.964	39.995	15	206	<0.0001
16	Hengchun, Pingtung	43.935	21.498	3	182	<0.0001

**Table 3:** Wind direction and degree.

Category	Degree	Direction
1	348.75–11.25	N
2	11.25–33.75	NNE
3	33.75–56.25	NE
4	56.25–78.75	ENE
5	78.75–101.25	E
6	101.25–123.75	ESE
7	123.75–146.25	SE
8	146.25–168.75	SSE
9	168.75–191.25	S
10	191.25–213.75	SSW
11	213.75–236.25	SW
12	236.25–258.75	WSW
13	258.75–281.25	W
14	281.25–303.75	WNW
15	303.75–326.25	NW
16	326.25–348.75	NNW

**Table 4:** Descriptive statistics of two daily weather covariates.

Site	City/County	PRE			TEM			
		Mean	Std	Max	Mean	Std	Min	Max
1	Shilin, Taipei	4.70	14.40	202.0	23.94	5.32	8.10	33.10
2	Taoyuan, Taoyuan	5.02	15.28	175.0	22.25	5.56	6.90	32.40
3	Dongqu, Hsinchu	4.03	14.64	260.0	23.41	5.35	7.60	31.80
4	Miaoli, Miaoli	3.73	12.53	151.0	23.32	5.35	7.50	31.60
5	Xitun, Taichung	4.22	15.23	179.0	23.80	5.03	7.90	31.60
6	Fengyuan, Taichung	5.17	19.28	279.0	23.20	4.84	6.40	30.80
7	Changhua, Changhua	4.54	15.97	164.5	23.38	4.63	7.80	31.20
8	Douliu, Yunlin	4.67	19.41	415.0	23.70	4.64	8.20	31.30
9	Xingang, Chiayi	3.87	16.43	332.5	23.93	4.71	9.00	31.20
10	West, Chiayi	4.85	19.32	417.0	24.35	4.59	9.20	31.70
11	West Central, Tainan	5.00	21.71	373.0	24.97	4.44	9.70	31.60
12	Xinying, Tainan	4.62	20.50	451.5	24.33	4.57	9.00	31.50
13	Zuoying, Kaohsiung	4.62	19.24	283.5	24.97	4.03	10.20	30.90
14	Qiangin, Kaohsiung	5.06	21.02	291.5	25.32	3.85	10.90	30.30
15	Pingtung, Pingtung	6.44	24.92	357.0	25.14	3.57	11.00	31.20
16	Hengchun, Pingtung	5.42	20.18	326.0	25.91	3.12	15.50	31.00

**Table 5:** Accuracy based on two forecast levels.

Site	City/County	Accuracy (%)			
		ARX-GARCH	AR Logistic	SVM	NNARX
1	Shilin, Taipei	95.20	95.20	95.47	95.47
2	Taoyuan, Taoyuan	95.20	95.44	96.53	96.53
3	Dongqu, Hsinchu	93.60	93.16	97.07	93.07
4	Miaoli, Miaoli	96.00	96.71	96.80	97.33
5	Xitun, Taichung	86.13	87.85	90.93	89.07
6	Fengyuan, Taichung	88.53	89.62	88.27	89.07
7	Changhua, Changhua	91.73	91.20	94.67	94.13
8	Douliu, Yunlin	78.13	81.52	84.27	82.93
9	Xingang, Chiayi	83.47	86.58	85.60	84.27
10	West, Chiayi	81.87	85.32	85.60	88.27
11	West Central, Tainan	80.53	83.04	83.20	82.93
12	Xinying, Tainan	84.00	87.85	87.20	86.13
13	Zuoying, Kaohsiung	78.93	82.67	81.60	79.73
14	Qianjin, Kaohsiung	76.80	82.40	83.47	82.13
15	Pingtung, Pingtung	73.07	78.13	82.93	78.40
16	Hengchun, Pingtung	97.60	98.23	97.07	97.07

**Table 6:** Accuracy based on four forecast levels.

Site	City/County	Accuracy (%)			
		ARX-GARCH	AR Logistic	SVM	NNARX
1	Shilin, Taipei	95.47	95.20	95.47	95.47
2	Taoyuan, Taoyuan	96.80	96.80	96.53	93.03
3	Dongqu, Hsinchu	93.33	93.60	97.07	93.07
4	Miaoli, Miaoli	97.07	97.07	96.80	97.33
5	Xitun, Taichung	86.67	89.07	90.40	88.80
6	Fengyuan, Taichung	88.27	88.80	88.27	88.80
7	Changhua, Changhua	93.87	94.67	94.67	94.13
8	Douliu, Yunlin	76.27	82.13	82.13	81.07
9	Xingang, Chiayi	84.00	85.07	85.60	84.27
10	West, Chiayi	82.93	85.33	85.07	88.00
11	West Central, Tainan	82.13	83.73	82.67	82.67
12	Xinying, Tainan	86.13	87.20	86.67	85.87
13	Zuoying, Kaohsiung	75.73	78.13	76.80	75.47
14	Qianjin, Kaohsiung	75.20	80.53	82.40	80.53
15	Pingtung, Pingtung	70.13	74.40	78.93	74.93
16	Hengchun, Pingtung	97.07	96.80	97.07	96.80
	Average (%)	86.32	88.03	88.53	87.52
	Std (%)	8.825	7.182	7.000	7.234
	CV	0.1022	0.0816	0.0790	0.0826

## Data Availability

The daily AQI values supporting the reported results can be found at https://data.epa.gov.tw/dataset/aqx_p_488, (accessed on 29 June 2021). The daily weather variables are available from https://e-service.cwb.gov.tw/HistoryDataQuery/, (accessed on 29 June 2021).

## Abbreviations

The following abbreviations are used in this manuscript:

AQI	Air quality index
AR logistic regression	Autoregressive multinomial logistic regression
ARX	Autoregression with exogenous variables
ARCH	Autoregressive conditional heteroskedasticity
GARCH	Generalized autoregressive conditional heteroskedasticity
NNARX	Neural network autoregression with exogenous variables
SVM	Support vector machine
